# The role of inflammasomes as central inflammatory hubs in *Mycobacterium tuberculosis* infection

**DOI:** 10.3389/fimmu.2024.1436676

**Published:** 2024-09-11

**Authors:** Sebastian J. Theobald, Tony A. Müller, Dinah Lange, Katharina Keck, Jan Rybniker

**Affiliations:** ^1^ Department I of Internal Medicine, University of Cologne, Cologne, Germany; ^2^ Center for Molecular Medicine Cologne (CMMC), University of Cologne, Cologne, Germany; ^3^ German Center for Infection Research (DZIF), Partner Site Bonn-Cologne, Cologne, Germany

**Keywords:** *Mycobacterium tuberculosis*, inflammasome, tuberculosis, drug resistance, interleukin-1, NLRP3 inflammasome, AIM2 inflammasome, gasdermin

## Abstract

*Mycobacterium tuberculosis* (*Mtb*) infection represents a global health problem and is characterized by formation of granuloma with a necrotic center and a systemic inflammatory response. Inflammasomes have a crucial role in the host immune response towards *Mtb*. These intracellular multi-protein complexes are assembled in response to pathogen-associated molecular patterns (PAMPs) or danger-associated molecular patterns (DAMPs). Inflammasome platforms activate caspases, leading to the maturation of the proinflammatory cytokines interleukin (IL)-1 and 18 and the cleavage of gasdermin D (GSDMD), a pore-forming protein responsible for cytokine release and pyroptotic cell death. Recent *in vitro* and *in vivo* findings have highlighted the importance of inflammasome signaling and subsequent necrotic cell death in *Mtb*-infected innate immune cells. However, we are just beginning to understand how inflammasomes contribute to disease or to a protective immune response in tuberculosis (TB). A detailed molecular understanding of inflammasome-associated pathomechanisms may foster the development of novel host-directed therapeutics or vaccines with improved activity. In this mini-review, we discuss the regulatory and molecular aspects of inflammasome activation and the associated immunological consequences for *Mtb* pathogenesis.

## 1 Introduction

Despite global control efforts, tuberculosis (TB) remains a leading cause of death worldwide with approximately 10.6 million new cases and 1.4 million deaths in 2022 (1). The causative agent of the disease, *Mycobacterium tuberculosis* (*Mtb*), is mainly transmitted via aerosols leading to pulmonary infection as well as various extrapulmonary manifestations (2). The course of the disease is characterized by long-lasting persistence, pointing towards various pathogenic strategies that have evolved in order to evade control by the immune system (3). As an intracellular pathogen, *Mtb* induces notable alterations in the cell death mechanisms of alveolar macrophages, a highly relevant host cell of this pathogen (4). Macrophage phagocytosis can trigger necrotic cell death, resulting in inflammatory tissue damage and bacterial spread.

There is evidence that innate immune responses activated by *Mtb* are regulated by large multimeric protein complexes called inflammasomes. These consist of a cytosolic sensor, an adaptor protein ASC (apoptosis-associated speck-like protein containing a CARD), and an effector caspase pro-caspase-1 (5–7). Inflammasome assembly is triggered by the recognition of exogenous or endogenous danger signals by various sensor molecules. Subsequent cleavage and activation of the pro-caspase-1 results in the secretion of the pro-inflammatory cytokines interleukin 1β (IL-1β) and interleukin 18 (IL-18), as well as the induction of pyroptosis. Pyroptosis is a pro-inflammatory form of programmed cell death, mediated by the cleavage of a pore-forming protein known as gasdermin D (GSDMD).

Several types of inflammasomes have been described, including the nucleotide-binding oligomerization domain (NOD), leucine-rich repeat (LRR)-containing protein (NLR) family members NLRP1, NLRP3 and NLRC4, as well as the proteins absent in melanoma 2 (AIM2) and pyrin. These inflammasome types are mainly characterized by the specificity of the sensor protein towards a certain stimulus (6). For instance, the AIM2 inflammasome acts as a sensor for (cytosolic) double-stranded DNA and the NLRC4 inflammasome responds to bacterial flagellin and type III secretion system components (8–11). In contrast, the NLRP3 inflammasome responds to a diverse set of microbial, endogenous and environmental stimuli. Priming of the inflammasome induces transcriptional upregulation of NLRP3 complex members as well as posttranslational modifications. This process is initiated by cytokine receptors or Toll-like receptors (TLRs) after recognition of pathogen-associated molecular patterns (PAMPs) or damage-associated molecular patterns (DAMPs), ultimately inducing nuclear factor-κB (NF-κB) activation (12). The second step towards full activation of the NLRP3 inflammasome can be triggered by a large variety of unrelated signals such as ATP or ion influx (12–14).

## 2 *Mtb* mediated modulation of the NLRP3 inflammasome *in vitro and ex vivo*

**Figure 1:**
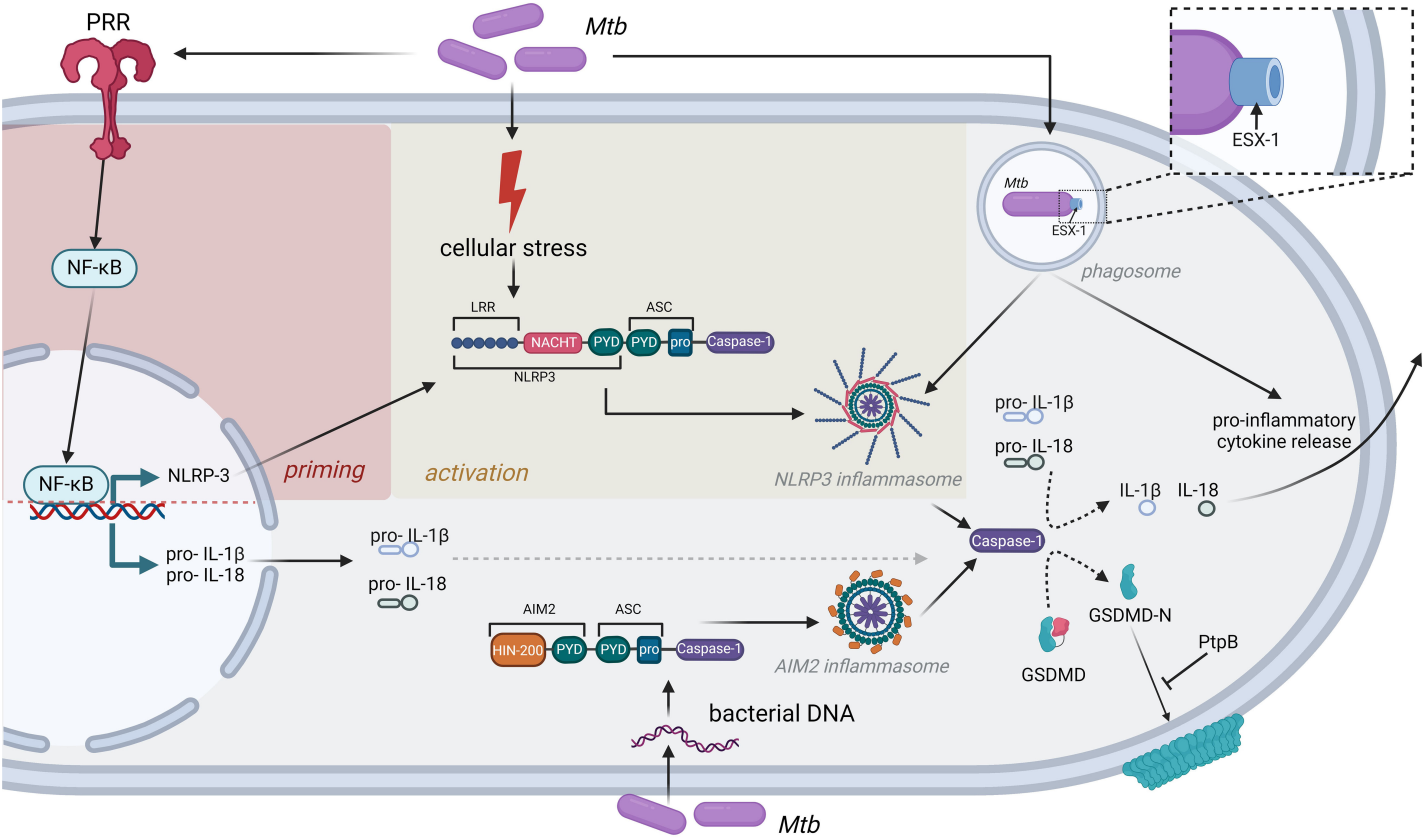
Inflammasomes as central mediators of inflammatory cytokine release after *Mtb* infection. Detection of *Mtb* by PRR induces cellular stress, leading to priming and activation of the NLRP3 inflammasome. In addition, *Mtb* DNA can be directly recognized by the AIM2 inflammasome. Both inflammasomes induce caspase-1 activation and subsequent release of inflammatory cytokines like IL-1β or IL-18. *Mtb* has evolved strategies to interfere with this immune response, exemplified by the bacterial PtpB phosphatase, which interferes with GSDMD binding to the cell membrane, or the ESX-1 secretion system that is strictly required to induce NLRP3 inflammasome formation.

Several reports indicate that *Mtb* infection of phagocytes *in vitro* or *ex vivo* leads to NLRP3 inflammasome activation, in combination with hallmarks of pyroptosis, including caspase-1 and GSDMD cleavage and the secretion of IL-1β and IL-18. This induction of NLRP3 inflammasome signaling by *Mtb* has been associated with multiple cell types including macrophage cell lines as well as murine and human-derived primary cells (THP-1 macrophages, monocyte-derived macrophages, bone marrow derived macrophages (BMDMs), bone marrow derived dendritic cells (BMDCs), murine retinal pigment epitheliums and primary murine microglial cells) (15–22). Using chemical inhibition and knock-out cell lines, we and others found several lines of evidence that key inflammasome components such as NLRP3, caspase-1 and GSDMD are required to mediate *Mtb-*induced pyroptotic cell death and the release of IL-1β and IL-18. In addition, several reports linked NLRP3 inflammasome activation with a functional ESX-1 secretion system (17–19, 22). This key virulence factor of *Mtb* secretes a pore forming protein (EsxA) into the cytosol of infected host cells and chemical inhibition of ESX-1 strongly impairs IL-1β release (23). However, the molecular mechanism by which EsxA or other co-secreted effector proteins mediate NLRP3 inflammasome activation is not known. Interestingly, EST-12, another secreted *Mtb* effector protein, binds to the cellular receptor RACK-1 leading to NLRP3/caspase-1/GSDMD mediated pyroptosis *in vitro* and *in vivo.* Consequently, an *Mtb* strain lacking EST-12 exhibited increased infectivity in the murine model, indicating that inflammasome activation can be protective in early stages of the infection (24). However, EST-12 is not secreted in an ESX-1 dependent manner, indicating the potential involvement of other secretory machineries of *Mtb* in NLRP3 inflammasome activation. Intriguingly, Chai et al. recently demonstrated that *Mtb* interferes with cell death mechanisms by secreting the phospholipid phosphatase (PtpB), which dephosphorylates plasma membrane phosphoinositides by binding to ubiquitin. PtpB enzymatic activity impairs binding of GSDMD to the plasma membrane subsequently leading to inhibition of pyroptosis (25). In line with this finding, we were able to show that IL-1β secretion of *Mtb*-infected macrophages is independent of GSDMD, but not NLRP3, indicating that alternative pore forming mechanisms are used to circumvent PtpB-dependent abrogation of GSDMD pore formation (17) (**Figure 1**).

While some of the studies discussed above point towards a role for *Mtb* in inducing inflammasome activation *in vitro*, some reports indicate that certain *Mtb* proteins might also have a suppressive effect on NLRP3 inflammasome activation. Rastogi et al. reported that *Mtb* infection of BMDMs could inhibit NLRP3 inflammasome activation in an ESX-1 independent mechanism. However, deletion of the bacterial phosphokinase PknF reversed this phenotype due to increased intracellular ROS production (26). Another study identified the bacterial protein PE12 as an inhibitor of NLRP3 inflammasome formation by PE12 binding to TLR-4 and subsequently silencing downstream signaling cascades (27). These *in vitro* findings require confirmation in suitable *in vivo* infection models that mirror necrotic granuloma formation observed in tissues of infected humans.

## 3 The role of IL-1β secretion and NLRP3 inflammasome activation in *in vivo* infection models

Despite numerous *in vitro* studies deciphering inflammasome-associated cytokine release and cell death pathways such as pyroptosis in the context of *Mtb* infection, there are contradictory data regarding the *in vivo* relevance of these findings.

Using mouse models of *Mtb* infection, several reports illustrate that knock-out of *Nlrp3* or *caspase-1* has little impact on survival, bacterial burden or the inflammatory responses (19, 28–30), whereas *Pycard* (the gene coding for ASC) knock-out mice show decreased survival associated with impaired granuloma formation (28). Recently, Stutz et al. reported that non-apoptotic functions of caspase-8, as well as caspase-1 or GSDMD do not contribute to the control of *Mtb* growth or *Mtb*-specific immune responses *in vivo*. In contrast, *Mtb* infection of their *Bax*/*Bak* knock-out model led to upregulation of multiple cytokines in lung tissue including IL-1β together with exacerbated lung lesions and higher bacterial loads, again indicating that GSDMD plays a minor role in secretion of this cytokine (31). Recently, Thomas et al. reported higher susceptibility towards *Mtb* infection in mice lacking phagocyte oxidase (Cybb) and caspase-1, however in their single knock-out for *caspase-1* they were not able to show any differences compared to wild-type mice (30). In contrast, Chai et al. recently elucidated, as mentioned above, the role of PtpB in abrogation of GSDMD binding to the plasma membrane. In their work, they proposed that inflammatory cytokine–mediated granuloma formation is mediated by GSDMD and further showed that PtpB is an essential component of *Mtb* to evade host GSDMD-mediated immune responses *in vivo* (25).

In a C3HeB/FeJ mouse model, which better reflects human granuloma formation compared to conventional mouse models, Lovey et al. were able to show that alveolar macrophages strongly upregulate genes such as NLRP3, IL-1 α/β, TNF and several CCL`s and CXCL`s. Using an IL-1R1 blocking antibody, they demonstrated that bacterial control, *Mtb* pathogenicity and *Mtb*-specific T cell priming is dependent on IL-1R1 signaling (32). In line with these findings, we detected increased expression of NLRP3 in lung granuloma tissue derived from IL-13_tg_ mice, another mouse model exhibiting human-like granuloma structures (17). Similar to the mentioned studies, Mayer-Barber et al. identified a crucial role for IL-1α and IL-1β, the proteins ultimately secreted after inflammasome activation, in conferring host resistance. Furthermore, they detected higher expression levels of both cytokines in lung macrophages and dendritic cells upon *Mtb* infection (33). The same group confirmed the importance of IL-1β, IL1R1 and type I interferons for *Mtb* pathology, in particular deciphering the immune crosstalk between both signaling pathways (34). Furthermore, the researchers propose that both targets are suitable for host-directed therapy approaches to control the outcome of TB (34). Taken together, there is clear evidence that IL-1β and the IL1R1 play important roles in *Mtb* pathogenicity *in vivo*, however, no consensus on the *in vivo* relevance of NLRP3 inflammasome components (NLRP3, caspase 1 and GSDMD) has been be achieved so far. Importantly, future research should focus on animal models that better reflect human granuloma formation and immunology.

## 4 The role of the AIM2 inflammasome during *Mtb*-infection

The first report highlighting the role of the AIM2 inflammasome during *Mtb* infection was published in 2012 by Saiga and colleagues (35). They detected increased susceptibility of *Aim2^-/-^* mice to intratracheal infection, which they related to decreased caspase-1 cleavage as well as lower IL-1β and IL-18 levels in the lungs. *Mtb*-antigen-specific T cell responses were also severely reduced in CD4^+^ T cells derived from *Aim2^-/-^* mice. *In vitro* data similarly revealed that depletion of AIM2 or ASC in murine macrophage cell lines diminishes IL-1β release following infection with *Mtb* or *Mycobacterium bovis* (36, 37). In addition, *Mtb* genomic DNA was confirmed to bind and activate AIM2 (35). Yan et al. revealed in *in vitro* experiments, that recognition of *Mtb* genomic DNA by AIM2 inhibits stimulator of IFN genes (STING) via interaction with ASC. This mechanism induces overreactive IFN-β and depressive IFN-γ responses *in M. bovis*-BCG infected *Aim2-/-* mice, translating into higher bacterial burden and more severe pathology (38). However, it remains unclear whether these results are of relevance for a better understanding of *Mtb* pathogenicity as the *M. bovis* -BCG vaccine strain lacks important virulence factors such as the ESX-1 secretion system, which is also crucial for the activation of AIM2 inflammasome components (39). *Ex vivo* studies using *Aim2*-deficient murine BMDMs or BMDCs have produced some contrasting results. *Mtb* as well as virulent *M. bovis* were reported to be capable of inducing AIM2 expression in BMDCs (36, 40). However, the impact of the increased expression remains unclear, as neither deficiency nor siRNA-medicated knockdown of *Aim2* had an influence on IL-1β release in *Mtb*-infected BMDMs (39, 40). In a further step, Shah et al. were addressing, whether *Mtb* could actively inhibit AIM2 inflammasome activation originating from other sources, such as simultaneous infection of BMDMs with *Mycobacterium smegmatis*. Indeed, they found that *Mtb* is able to diminish the AIM2-dependent IL-1β production caused by this non-pathogenic fast-growing mycobacterium (39). In line with the host-protective role of AIM2 in *in vivo* experiments, it was also reported that IL-1β production is strongly reduced in *Aim2^-/-^*/*Nlrp3^-/-^* BMDMs infected with different clinical *Mtb* strains. However, it was not addressed, how much of this effect can be attributed to the individual inflammasome types (41).

Together, these studies indicate that the exact role of the AIM2 inflammasome pathway in *Mtb* infection remains not fully understood and that experimental findings may be influenced by the nature of the infected host cell (mouse versus human) or the mycobacterial strain under investigation. Some data point towards *Mtb* inhibition of AIM2 to be a potential evolutionary mechanism allowing immune evasion of the pathogen.

## 5 Concluding remarks and discussion

Although some light has been shed on *Mtb* host-pathogen interactions in recent years, the precise role of inflammasomes in this context is not fully understood. Here we systematically reviewed the current literature on the impact of NLRP3 and AIM2 inflammasome activation for *Mtb* pathogenicity. In summary, there is strong evidence from *in vitro* experiments that both inflammasome types are being modulated in *Mtb* infected macrophages, which leads to secretion of several pro-inflammatory cytokines as well as pyroptotic cell death (**Figure 1**). Consistent with this notion, there have been reports of *NRLP3* gain-of-function variants in TB patients that confer some degree of resistance towards *Mtb* infection (42, 43). Interestingly, inflammasome activation is also increased in tuberculosis-associated immune reconstitution inflammatory syndrome (TB-IRIS), a hyperinflammatory state that is frequently observed in patients co-infected with HIV (44). In contrast, conventional knock-out mouse models failed to demonstrate any impact of NLRP3 or caspase-1 in *Mtb* pathogenicity, whereas mice deficient for AIM2 or PYCARD seemed to be more susceptible to *Mtb* infection. However, it remains doubtful whether results from these mouse models can be translated to *Mtb* infection in humans, as necrotic granuloma formation is not observed in these mice. A potential consequence might be that the immunological signaling pathways triggered by *Mtb* in these models differ from the human setting. Therefore, we and others have explored mouse models that reflect the human situation more closely, such as C3HeB/FeJ mice and IL-13_tg_ mice, where NLRP3 and IL-1 signaling seems to play a more relevant role, which needs to be confirmed in future studies. At this point, it is important to note that the usage of more human-like *in vivo* models is essential to decipher the importance of inflammasome signaling in TB. Despite the contradictory results regarding the role of NLRP3 and AIM2, murine and human *in vitro* and murine *in vivo* studies consent that IL-1α/β and IL1R1 play an important role in *Mtb* pathogenicity. The relevance of these pathways has particularly been highlighted by a report connecting IL-1 signaling with type 1 interferon signaling, which is known to be involved in *Mtb* immunobiology (45). Therefore, IL-1R1 blockade represents a potential candidate for host-directed therapy (HDT) approaches. The development of novel and potent HDTs for the treatment of TB is of particular importance with the emergence of more multi-drug resistant strains. In general, inflammasomes represent a highly interesting target for HDTs, as a pharmacological intervention would potentially inhibit the excessive secretion of pro-inflammatory cytokines and, secondly, suppress tissue damage and necrosis mediated by host cell death.

Along these lines, we and others have shown *in vitro* activity for the selective NLRP3 inflammasome inhibitor MCC-950, a small-molecule, which has been used in a clinical study for rheumatoid arthritis (46). MCC-950 potently inhibits *Mtb*-induced cell death and further abrogates IL-1β and IL-18 secretion (17, 22). Similar results have been obtained for the small-molecule Vx-765, which blocks caspase-1 (17, 22). Further specific inhibitors of the inflammasome pathway are currently under development (47). However, for all potential inhibitors, *in vivo* efficacy studies are missing. Notably, we believe that the clinical success of such HDTs relies on a personalized treatment approach, which identifies the ideal time-point for the intervention and reduces potential side effects.

For this, a better understanding of the exact timing of inflammasome and IL-1 signaling during *Mtb* infection is crucial (i) to gain new insights into the immunopathology of *Mtb* during acute and chronic disease in suitable *in vivo* and *ex vivo* models and (ii) to identify patients that may benefit from inhibitors within host directed therapies.
